# Model-based strategy for cell culture seed train layout verified at lab scale

**DOI:** 10.1186/1753-6561-9-S9-P44

**Published:** 2015-12-14

**Authors:** Simon Kern, Oscar B Platas, Martin Schaletzky, Volker Sandig, Björn Frahm, Ralf Pörtner

**Affiliations:** 1 Institute of Bioprocess and Biosystems Engineering, Hamburg University of Technology, Hamburg, D-21073, Germany; 2 Biotechnology & Bioprocess Engineering, Ostwestfalen-Lippe University of Applied Sciences, Lemgo, D-32657, Germany; 3 ProBioGen AG, Berlin, D-13086, Germany

## Background

Production of biopharmaceuticals for diagnostic and therapeutic applications with suspension cells in bioreactors requires a seed train up to production scale [1]. For the first steps - the transitions between T-flasks, tubes, roller bottles, shake flasks, stirred bioreactors or single-use reactors - the experimental effort to lay-out these steps is high. At the same time it is known that the first cultivation steps have a significant impact on the success or failure in production scale. A software tool has been developed which provides possibilities for simulation, analysis and design of seed trains [2]. Tool structure:

• A kinetic model. In this case a simple unstructured model where kinetic parameters can be obtained from a few experiments only.

• A Nelder-Mead-algorithm to determine model parameters.

• A developed MATLAB software tool able to determine optimal points in time or viable cell concentrations for transfer into the next scale.

The successful application for the cell line (AGE1.HN*_AAT_*, ProBioGen AG) has been shown previously [3]. Here the tool was tested for a suspendable CHO cell line.

## Materials and methods

The cell line CHO-K1 has been grown in chemically defined TC-42 medium (TeutoCell AG, Bielefeld, Germany), 4 mmol L-1 glutamine.

Data for parameter identification for the kinetic mode were determined in shake flask cultures. The seed train steps were: 1. culture tube (0.0025 L), 2. shake flask (0.070 L), 3: Labfors 5 Cell (2 L).

## Results

**Figure 1:**
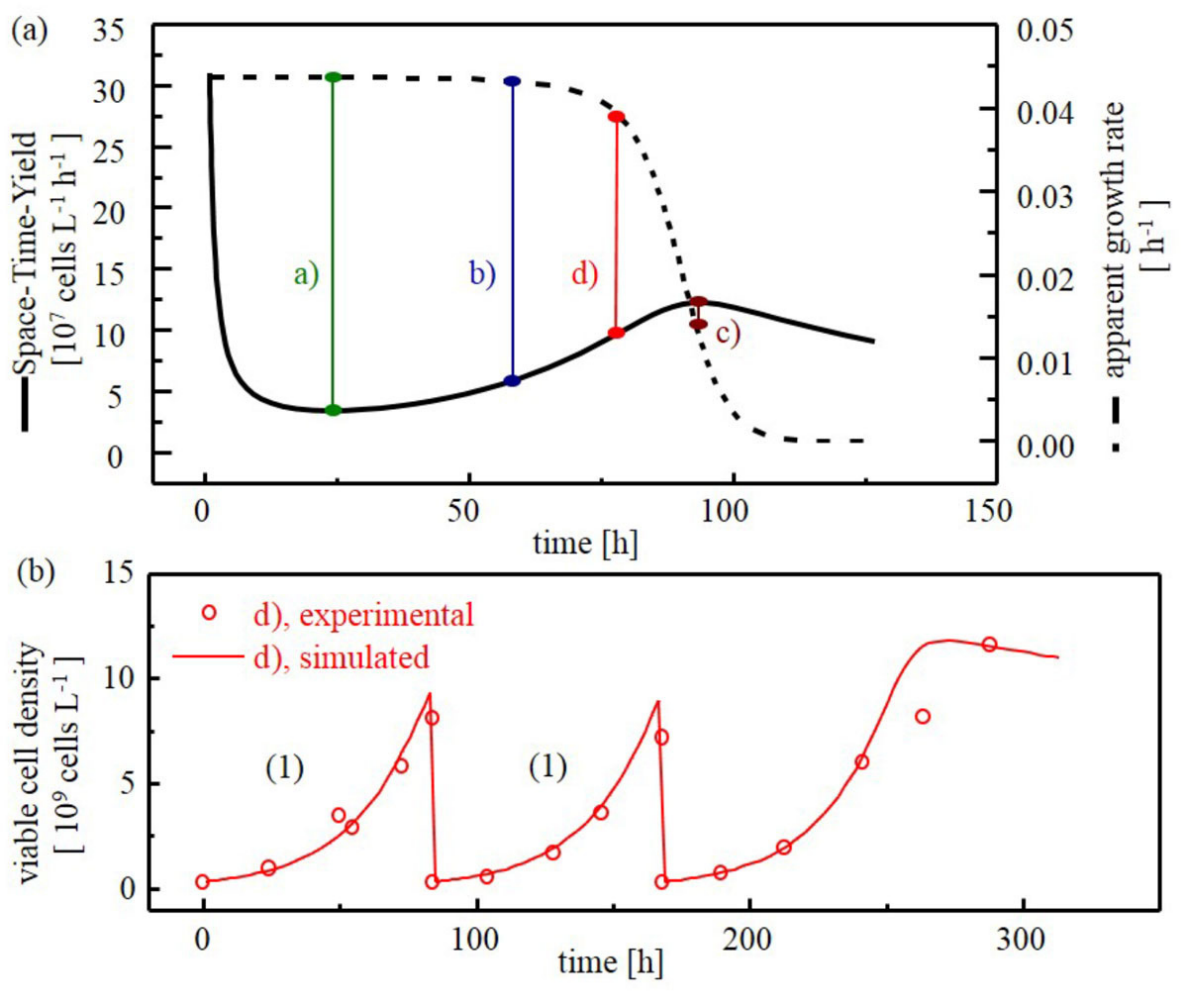
**(A) **simulated courses of Space-Time-Yield (STY) and apparent growth rateover time exemplarily for one scale: a) point in time of minimal STY, b) average value of a) and c) as a cell passaging criterion, c) point in time of maximal STY as a cell passaging criterion, d) average of time at maximal STY and time at 0.9·µ_max_ as a cell passaging criterion. **(B) **Seed train for CHO-K1 - simulated and experimental courses of viable cell density over time. Passaging of cells at the points in time calculated using average of time at maximal STY and time at 0.9·µ_max_ (criterion d)).The seed train steps were: 1. culture tube (0.0025 L), 2. shake flask (0.070 L), 3: Labfors 5 Cell (2 L)

For the seed train first different optimization criteria were compared in silico (Fig. 1a). Finally, the average of time at maximal space-time-yield (STY) and time at 90% of maximal growth rate (0.9·µmax) was used as optimization criterion for cell transfer. The concept was tested successfully up to a 2 L scale for 3 scale-up steps (Figure 1b).

## Conclusions

The concept offers a simple and inexpensive strategy for design of seed train scale-up steps. The results for the lab scale steps show that the tool was able to perform a seed train optimization only on the basis of two batches, the underlying model and its parameter identification.

## Acknowledgements

The bioreactor (Labfors 5 Cell) was kindly provided by the company Infors AG, the suspendable cell line CHO-K1 by Prof. Thomas Noll, University of Bielefeld.

## References

[B1] Eibl R Eibl D Pörtner R Catapano G Czermak P Cell and Tissue Reaction Engineering Springer 2008 ISBN 978-3-540-68175-5

[B2] Kern S Platas-Barradas O Pörtner R Frahm B Model-based strategy for cell culture seed train layout verified at lab scale Cytotechnolpublished online: 21 March 2015, DOI 10.1007/s10616-015-9858-9 10.1007/s10616-015-9858-9 PMC4960151 25795469

[B3] Kern S Platas O Schaletzky M Sandig V Frahm B Pörtner R Model-based design of the first steps of a seed train for cell culture processes BMC Proceedings 2013 7 Suppl 6 P11 (4 December 2013)

